# Supplementary material for: Mast Cell Interaction with Foxp3+ Regulatory T Cells Occur in the Dermis after Initiation of IgE-Mediated Cutaneous Anaphylaxis
Source: Cells. 2022 Sep 29;11(19):3055. doi: 10.3390/cells11193055 (PMC9564058; doi:10.3390/cells11193055)
Supplement: Supplementary file 1 [file cells-11-03055-s001.zip › cells-1873037-supplementary.pdf]

Supplementary Material

Legends to supplementary Figures and Videos.

S1A

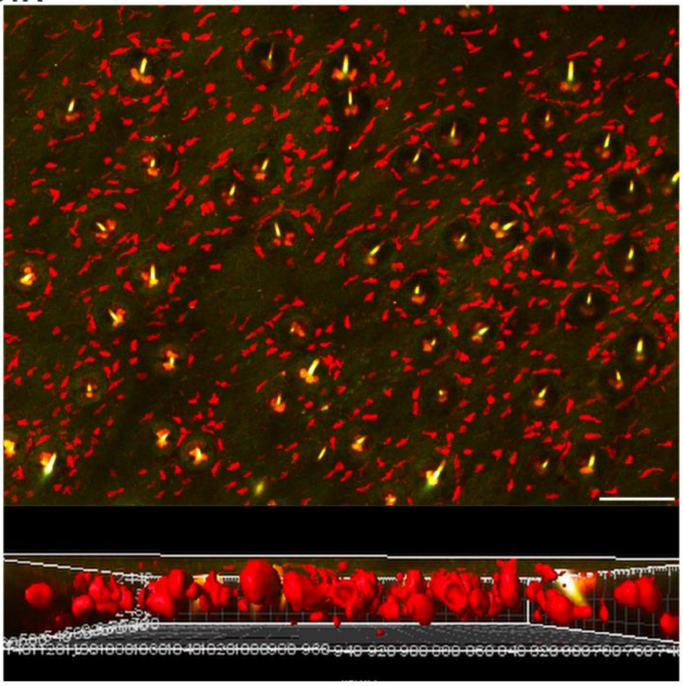

S1B

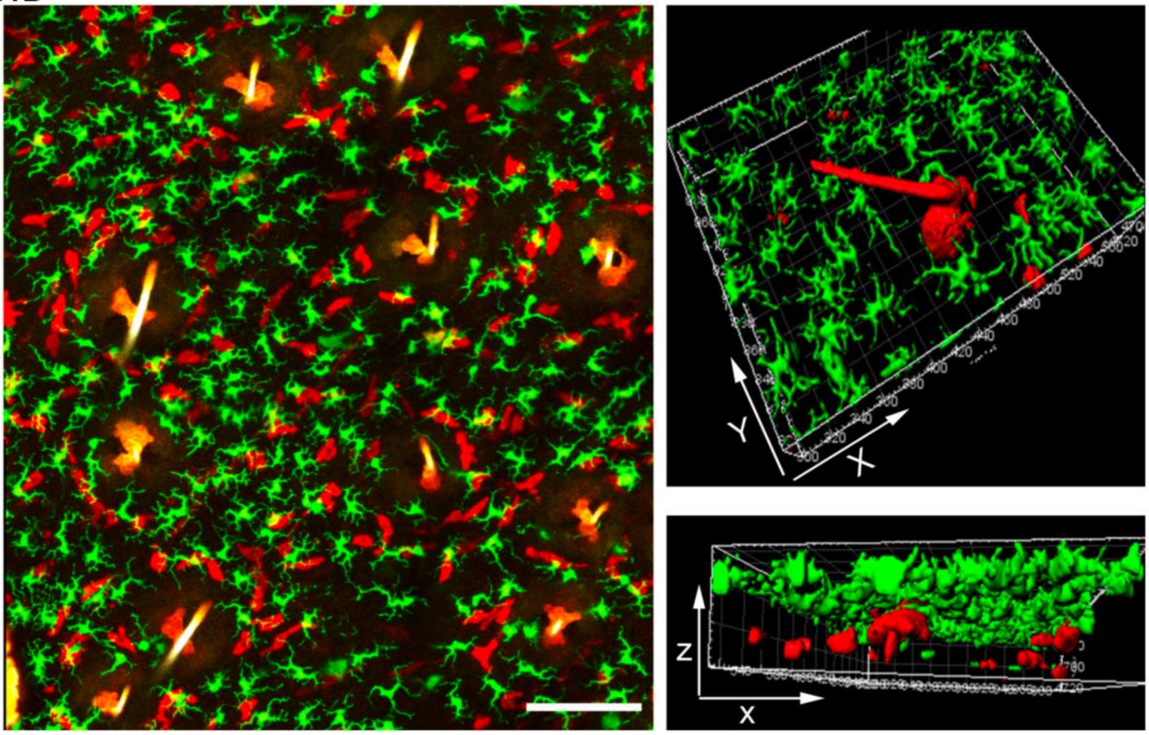

S1C

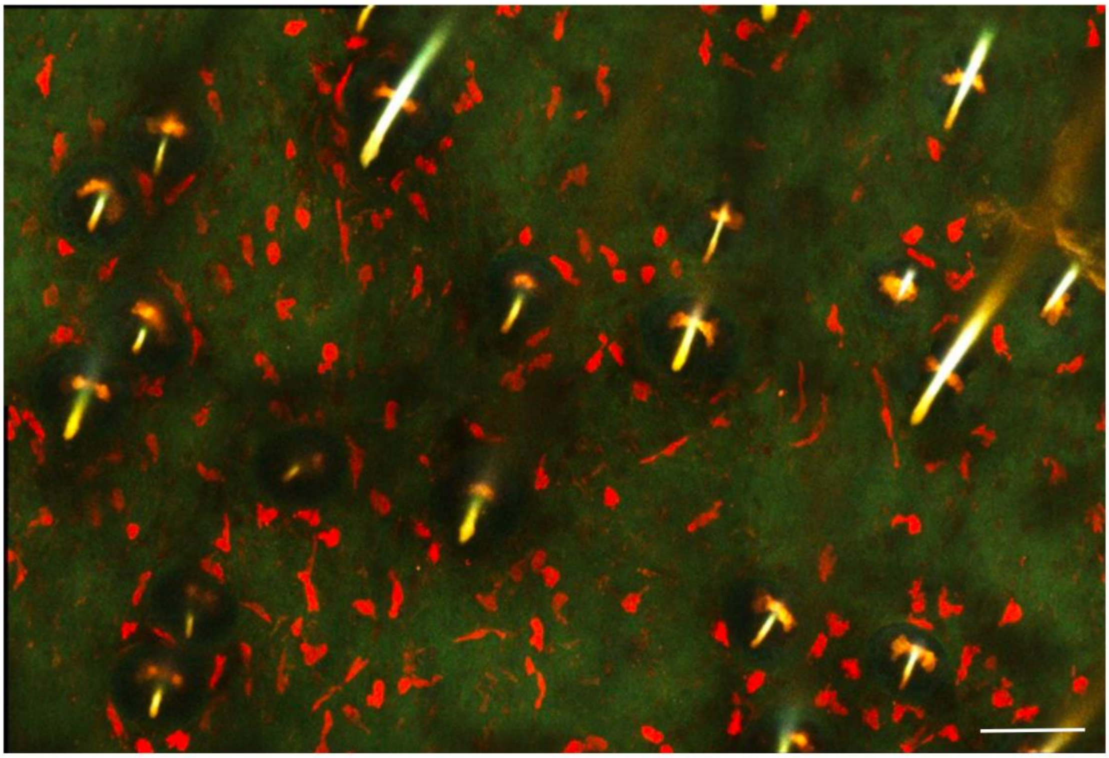

Figure S1. Characterization of mast cells and Langerhans cells in the skin of RMB and RMB-Langerin-eGFP mice (A) Mosaic image of 3D data stack with maximum Z projection over 45µm. Mast cells form a dense network of tdT^+ red fluorescent cells. Hair follicles and associated sebaceous glands give rise to autofluorescence emission in all channels visualized in yellow. Corresponding Imaris software based segmentation was used for 3D reconstruction of tdT^+ MCs (bottom part of panel A). **(B)** RMB mice were crossed with knock in Langerin-eGFP mice. Left panel : Mosaic image of 3D stack with maximum Z projection over 45µm were performed to include all cells in the epidermis and the dermis. Right panels : Imaris software based segmentation was used for 3D reconstruction of tdT^+ MCs and Langerin-$eGFP^+$ LCs indicating that MCs (in red) are located in the dermis below the layer of epidermal LCs (in green). **(C)** Distribution of MCs 3 months after DT-mediated conditional ablation in RMB mice**.** Mosaic image of 3D data stack with maximum Z projection over 45µm were

performed as in supplementary Figure 1A. Substantial levels of MCs are found although with uneven distribution. Scale bar **A), B)** and **C)** 100µm.

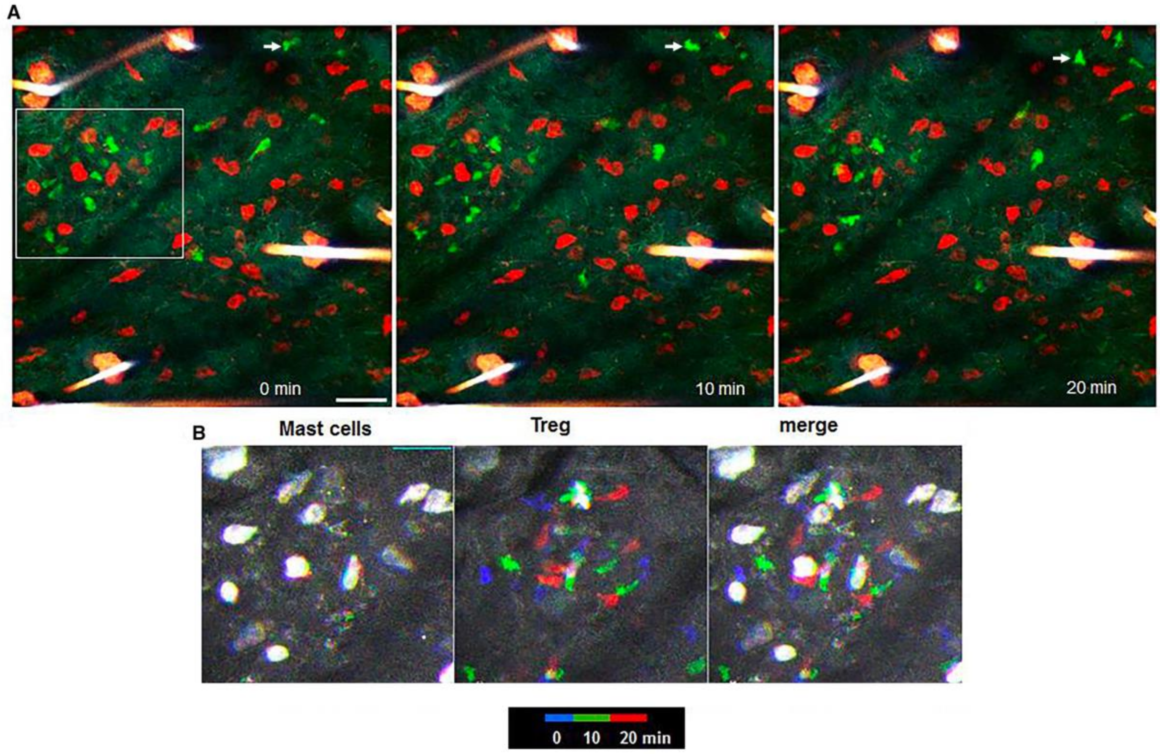

Figure S2. Visualization of MC-Treg interactions 2 to 3 hours after initiation of PCA reaction. Highly motile Tregs are recruited to the site of inflammation and reveal transient contacts with sessile MCs during PCA. Maximum Z projection of sequential 3D stacks images monitored at 0 (left panel), 10 (middle panel) and 20min (right panel) starting 2h after initiation of the PCA in live ear skin of an IgE- sensitized ear of RMB-Foxp3-eGFP mice 2h after challenge with Ag . An arrow follows a motile Treg. Scale bar 50µm. (**B)** Merged RGB images (0, 10 and 20 min) of the zoomed window defined in **(A)** show immobile merged MCs (left panel), mobile merged Tregs (middle panel) and the overlay of the two merged immobile MCs and motileTregs (right panel). MC, which are sessile appear in white, while motile Tregs are revealed by decomposed colors in this RGB representation of cell motility. These image data sets were compiled from dual fluorescence 4 dimensional data sets with Z

thickness of 45µm and 3µm Z step size monitored at 0, 10 and 20min starting 2h after initiation of the PCA.

Video S1. RMB-Foxp3eGFP mice, steady state: Motile Tregs and sessile MCs in live ear skin at homeostasis. The video shows 3D confocal data sets of tdT red fluorescent dermal MCs and some eGFP+ green fluorescent Tregs. MCs appear sessile over the entire data acquisition. The few Tregs are highly motile occasionally contacting MCs. The video shows a 60 min time frame with images taken every 4 min of the ear skin RMB-Foxp3eGFP mice.

Video S2. Confocal video images of MCs and Tregs in live ear skin of a control PBS- sensitized ear of RMB-Foxp3-eGFP mice after challenge with Ag. The video shows 3D confocal data sets of tdT red fluorescent MCs and eGFP+ green fluorescent Tregs. MCs appear sessile over the entire data acquisition. The few Tregs are highly motile occasionally contacting MCs. No significant changes compared to imaging at homeostasis are apparent. The video shows a 60 min time frame with images of the ear skin taken every 4 min and starting 2 h after i.v. injection of Ag into PBS-sensitized RMB-Foxp3eGFP mice.

Video S3. Confocal video images of MCs and Tregs in live ear skin of an IgE-sensitized ear of RMB-Foxp3-eGFP mice after challenge with Ag (DNP-HSA). The video represent 3D confocal data sets of tdT red fluorescent mast cells and eGFP+ green fluorescent Tregs. MCs appear sessile over the entire data acquisition. A large number of motile Tregs are visible many of them contacting MCs in a prolonged manner. It reveals the marked increase in Tregs and their interaction with sessile MCs.

The video shows a 60 min time frame with images of the ear skin taken every 4 min and starting 2 h after i.v. injection of Ag (DNP-HSA) into IgE-sensitized RMB-Foxp3eGFP mice.

Video S4. Confocal video images of sessile epidermal LCs in live ear skin of a control PBS-sensitized ear of RMB-Langerin-eGFP mice after challenge with Ag. The video shows 3D confocal data sets of eGFP+ green fluorescent LCs imaged after i.v. injection of Ag into control PBS-sensitized RMB-Langerin$_{eGFP}$ mice. The sentinel layer of LCs are sessile exhibiting constant sprouting of their dendrites. Some motile Langerin+ dermal DC can also be seen. In contrast to LCs they are motile. The video shows a 60 min time frame with images of the ear skin taken every 4 min and starting 3 h after i.v. Ag challenge.

Video S5. RMB-LangerineGFP mice, PCA Ag challenge: Confocal video images of sessile epidermal Langerhans cells in live ear skin in IgE sensitized mice after challenge with Ag (DNP-HSA). The video shows 3D confocal data sets of eGFP+ green fluorescent LCs imaged after i.v. injection of Ag into IgE-sensitized RMB-LangerineGFP mice. Within their whole epidermal layer, the sentinel LCs are sessile but exhibit constant sprouting of their dendrites. Some motile Langerin+ dermal DC can also be seen and are motile in contrast to LCs in the epidermis. The video shows a 60 min time frame with images of the ear skin taken every 4 min and starting 3 h after local i.v. injection of PBS into ear skin locally IgE-sensitized RMB-LangerineGFP mice.
